# The black fly salivary protein Simukunin inhibits complement activation through the lectin pathway protease MASP-2

**DOI:** 10.1016/j.jbc.2026.113529

**Published:** 2026-09-04

**Authors:** Michelle D. Jung, Molly E. Ring, Brian Gabriel Bonilla, Sundar Ganesan, Paola Carolina Valenzuela-Leon, Eric Calvo

**Affiliations:** 1 Laboratory of Malaria and Vector Research, National Institute of Allergy and Infectious Diseases, National Institutes of Health, Rockville, Maryland, USA; 2 Biological Imaging Section, Research Technologies Branch, National Institute of Allergy and Infectious Diseases, National Institutes of Health, Bethesda, Maryland, USA

**Keywords:** arthropods, saliva, blood feeding, complement, coagulation, vascular biology

## Abstract

Salivary secretions from blood-feeding arthropods are enriched with bioactive molecules that counteract host hemostatic and immune defenses, including blood coagulation, platelet aggregation, complement activation, and vasoconstriction. Although numerous salivary components have been identified and functionally characterized across hematophagous arthropods, their specific roles in complement inhibition in black flies remain insufficiently defined. Previously, it was discovered that Simukunin, a salivary protein from *Simulium vittatum*, inhibits procoagulant activity. Here, using surface plasmon resonance and enzymatic assays, we found that Simukunin also acts as a specific inhibitor of the complement lectin pathway (LP). Simukunin binds with high affinity to mannose-associated serine protease 2 (MASP-2), a serine protease crucial for LP activation, preferentially blocking cleavage of C2 over C4, resulting in reduced downstream membrane attack complex deposition. No interaction with other downstream complement proteins was found. Simukunin also inhibits the procoagulant activity of MASP-2 *in vitro.* Notably, pretreatment with Simukunin significantly improved survival rates in a murine model of lipopolysaccharide-induced sepsis. Overall, Simukunin emerges as a novel complement inhibitor and a potential candidate for therapeutic applications.

The human complement system is an important defense mechanism composed of multiple proteins that act together to produce an effective immune response. The complement system consists of three pathways: the classical pathway (CP), which is initiated by antigen–antibody complexes; the alternative pathway (AP), which is constitutively active; and the lectin pathway (LP), in which mannan-binding lectin (MBL) recognizes O-mannose residues on pathogen-associated carbohydrates or glycoproteins, leading to activation of MASP-1 and MASP-2. In particular, MASP-2 is a serine protease crucial for LP activation, which cleaves C4 and C2. The lectin and classical pathways use different recognition molecules but share key downstream effectors. Binding C1q to immune complexes activates C1r, which subsequently activates C1s. Like C1s, MASP-2 cleaves C4 and C2 to generate the C3 convertase. In addition, MASP-2 can cleave prothrombin, providing a mechanistic link between complement activation and coagulation, a property not attributed to C1s (1). Although activated by unique stimuli, all three complement pathways share the sequential formation of C3 convertases, activation of C5 convertases, and ultimately the assembly of the membrane attack complex (MAC, C5b-9), which is responsible for forming pores in target cell membranes, leading to cell lysis (2, 3, 4, 5). This system exerts strong selective pressure in host–pathogen interactions, driving female blood-feeding arthropods to evolve salivary inhibitors that block key steps of this cascade, thereby enabling efficient blood feeding required for reproductive success. Various inhibitors of the complement system have been identified. For instance, albicin from *Anopheles albimanus* associates directly with the alternative pathway C3 convertase (C3bBb), resulting in potent inhibition of AP activation both in the presence and absence of properdin (6, 7). Moreover, the binding of Salp20, a salivary protein from *Ixodes scapularis*, to properdin dissociates the active C3 convertase (C3bBbP), effectively inhibiting the AP and limiting C3 amplification (8, 9). Compared to other species, complement system inhibitors in black flies have not been extensively investigated. Because only female black flies feed on blood, their salivary glands are known to be a biologically relevant source of proteins involved in modulating host responses during blood feeding and pathogen transmission. Recently, we demonstrated that Guianensin, a serine protease inhibitor of the Kunitz family from *Simulium guianense*, exhibits anti-hemostatic and anti-inflammatory properties by inhibiting the LP through its interaction with MASP-2 (10, 11). Investigations into the North American female black fly *Simulium vittatum* have characterized its sialome (from the Greek word *sialo*, meaning saliva), defined as the totality of salivary gland transcripts and proteins, and further validated, through experimental studies, that there are salivary proteins that possess anti-hemostatic and immunomodulatory functions (12, 13, 14). However, the precise mechanisms by which these proteins act require further elucidation. Sequence alignment of Guianensin with the *S. vittatum* Kunitz protein, Simukunin, revealed 58% amino acid identity, demonstrating conservation of this salivary protein between the two species (15). Additional sequence comparisons of Guianensin have identified related Kunitz proteins in other blood-feeding vectors (10). Both proteins are members of the Kunitz family of proteins, which feature a highly conserved ∼60–amino acid domain typically stabilized by three disulfide bridges and functions as a serine protease inhibitor, suggesting a role in modulating host hemostatic or inflammatory responses during blood feeding (16). Simukunin was previously shown to exhibit anticoagulant activity through inhibition of factor Xa (FXa) (17). However, given its serine protease–inhibitory properties and similarity with Guianensin (10, 11), we hypothesized that Simukunin also targets MASP-2. Here, we demonstrate anticomplement activity of Simukunin in the LP, mediated by its direct interaction with MASP-2. Additionally, we did not find inhibitory activity against other proteases or components in the complement pathways. We also show that salivary gland extracts from female adult *S. vittatum* inhibit LP. Simukunin has a strong anti-inflammatory activity *in vivo,* significantly increasing mice survival in an LPS-induced generalized sepsis model. These findings highlight the therapeutic potential of black fly salivary proteins, which exhibit both anticoagulant and anticomplement activities.

## Results

### Salivary gland extract and simukunin inhibits complement activation *in vitro*

Female *S. vittatum* possesses a pair of salivary glands that contain proteins with anticoagulant, vasodilatory, and immunosuppressive activities (12, 13). Moreover, Simukunin, present in *S. vittatum* salivary gland extract, exhibits broad anti-protease activity and belongs to the same class as Guianensin. Based on these observations, we investigated whether *S. vittatum* salivary gland extract (SGE) and Simukunin could inhibit complement activation. Here, SGE amounts are expressed as salivary gland pair equivalents, representing the amount of extract corresponding to a given number of salivary gland pairs used in each assay. Complement activity was evaluated using a commercial kit that detects formation of the C5b-9 complex following activation of the classical (CP), lectin (LP), or alternative (AP) complement pathways. We show that SGE corresponding to one salivary gland pair and Simukunin at 1 μM significantly inhibited LP activation, with little effect on the CP or AP (Fig. 1, *A* and *B*). Dose–response analysis using increasing amounts of SGE pairs or increasing concentrations of Simukunin showed concentration-dependent inhibition of the LP, with an IC_50_ of 0.15 pairs and 3.3 nM respectively (Fig. 1, *C* and *D*).

**Figure 1:**
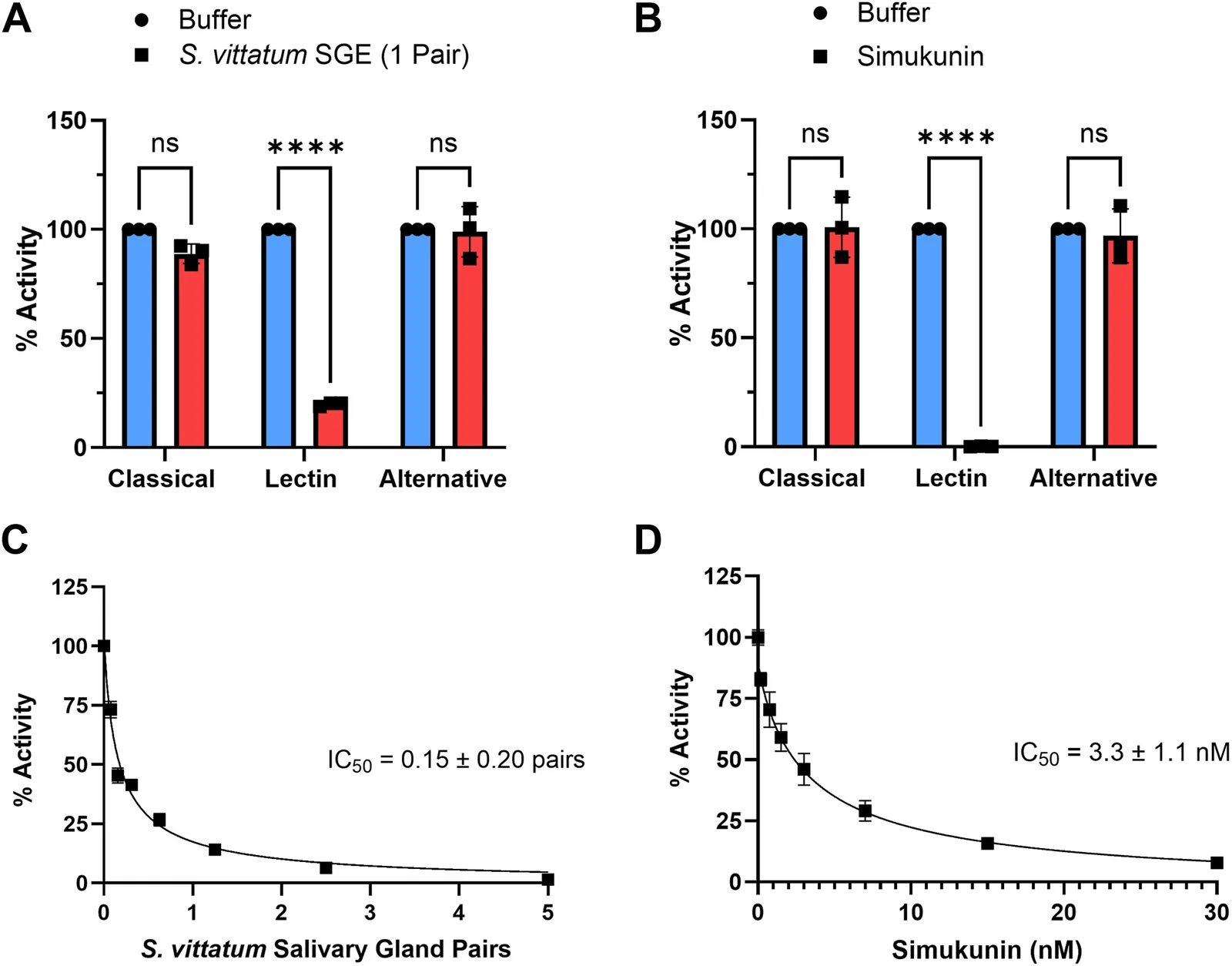
**Salivary gland extracts of *Simulium vittatum* and Simukunin inhibit complement activation.***A–B*, normal human serum (NHS) was preincubated with buffer or *S. vittatum* salivary gland extract (SGE; one pair) (*A*), or Simukunin (1 μM) (*B*), and applied to Wieslab complement assay strips specific for the lectin (MBL), classical (IgM), or alternative (LPS) pathways. After 60 min at 37 °C, complement activation was quantified by C5b-9 deposition and expressed as a percentage of the positive control (mean ± SD, n = 3 independent experiments). Statistical significance was determined using a two-tailed one-sample *t* test comparing SGE-treated samples with the normalized control value of 100%. No significant difference: ns; ∗∗∗∗*p* < 0.0001. *C–D*, to assess concentration-dependent inhibition of the lectin pathway, NHS was preincubated with increasing amounts of *S. vittatum* SGE (1–5 pairs) (*C*) or increasing concentrations of Simukunin (1–30 nM) (*D*). Lectin pathway activation was quantified by C5b-9 deposition and expressed as a percentage of the positive control (mean ± SD, n = 3 independent experiments). IC_50_ values were calculated by nonlinear regression using a four-parameter inhibitor–response model in GraphPad Prism 10.2.1. The corresponding standard errors (SE) of the fitted IC_50_ values are indicated in *panels C* and *D*.

### Simukunin binds to and inhibits MASP-2 catalytic activity

To determine the mechanism of which Simukunin inhibits the complement pathway, we performed surface plasmon resonance (SPR) with various proteases of the complement pathway over immobilized Simukunin. The SPR binding analysis shows that Simukunin binds specifically to the catalytic region of MASP-2 (Fig. 2*A*), an important serine protease in LP activation. No significant binding was detected for the other proteases in the complement pathways (Fig. 2*A*). Subsequently, we performed SPR kinetic experiments of Simulunin-MASP-2 interaction. Best fit was achieved with a global 1:1 model, yielding a *K*_*D*_ of 58.8 ± 14.6 nM (*k*_*on*_ of (4.8 ± 2.8) × 10^5^ M^−1^s^−1^ and *k*_*off*_ of (3.0 ± 2.3) × 10^-2^ s^*−1*^ (Fig. 2*B*). Moreover, we assessed the functional implications of this binding by examining the inhibitory effects Simukunin on the enzymatic activity of MASP-2. The results demonstrate that Simukunin significantly inhibited the amidolytic activity of MASP-2 in a dose-dependent manner, with a fitted *K*_*i*_ of 31.9 nM (Fig. 2*C*). Overall, these results demonstrate that Simukunin directly binds to MASP-2 with high affinity, supporting a mechanism in which Simukunin inhibits the LP.

**Figure 2:**
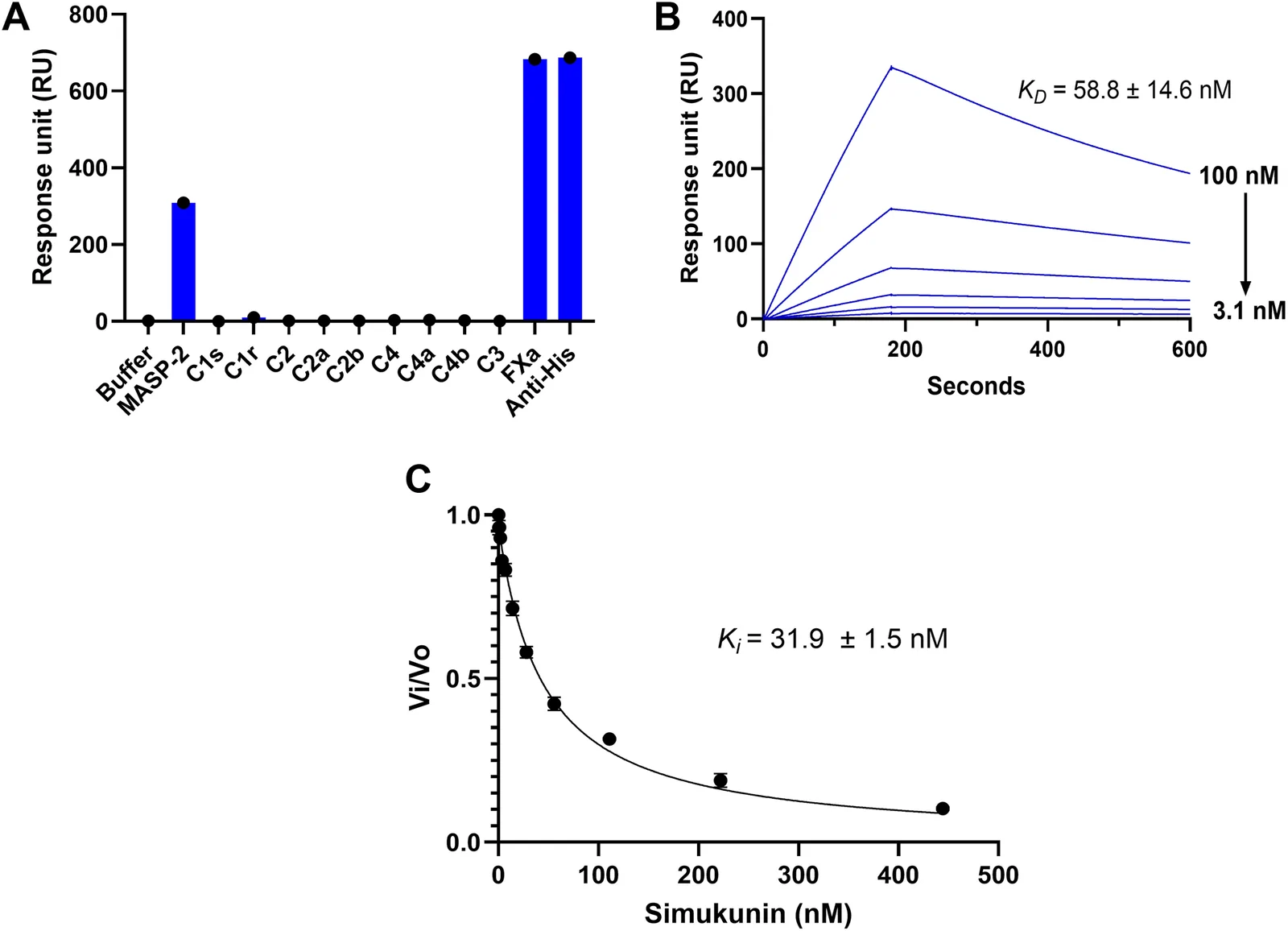
**Interaction of Simukunin with MASP-2.***A*, SPR-binding analysis showing the interaction between complement proteins (concentration 200 nM) and immobilized Simukunin (15 μg/ml). *B*, MASP-2 was injected at concentrations ranging from 3.1 to 100 nM over immobilized Simukunin for 180 s, and dissociation was monitored for 600 s. A global 1:1 binding model was applied to calculate kinetic parameters. *C*, MASP-2 (20 nM) was pre-incubated with increasing concentrations of Simukunin before addition of Z-Lys-SBzl and DTNB. Fitted parameters are reported as best-fit values ± standard error obtained from using a Morrison Ki model in GraphPad Prism 10.2.1.

### Simukunin inhibits cleavage of C2 by MASP-2

Upon activation of the LP, MASP-2 cleaves C2 and C4 to initiate downstream complement activation. To evaluate the effects of Simukunin binding to MASP-2, we incubated C2 or C4 with the recombinant catalytic region of MASP-2 and Simukunin. At different times, each reaction was stopped and analyzed with an SDS-PAGE gel followed by Coomassie blue staining. Simukunin had little effect on the cleavage of C4 by MASP-2, with only a modest reduction observed at early time points (15–20 min) (Figs. 3*A* and S2 (Densitometric analysis)). In contrast, Simukunin strongly inhibited the cleavage of C2 by MASP-2, as indicated by a marked reduction in the formation of C2 cleavage fragments (Figs. 3*B* and S2 (Densitometric analysis)). Together, these results show that Simukunin preferentially inhibits MASP-2 mediated cleavage of C2 compared to C4.

**Figure 3:**
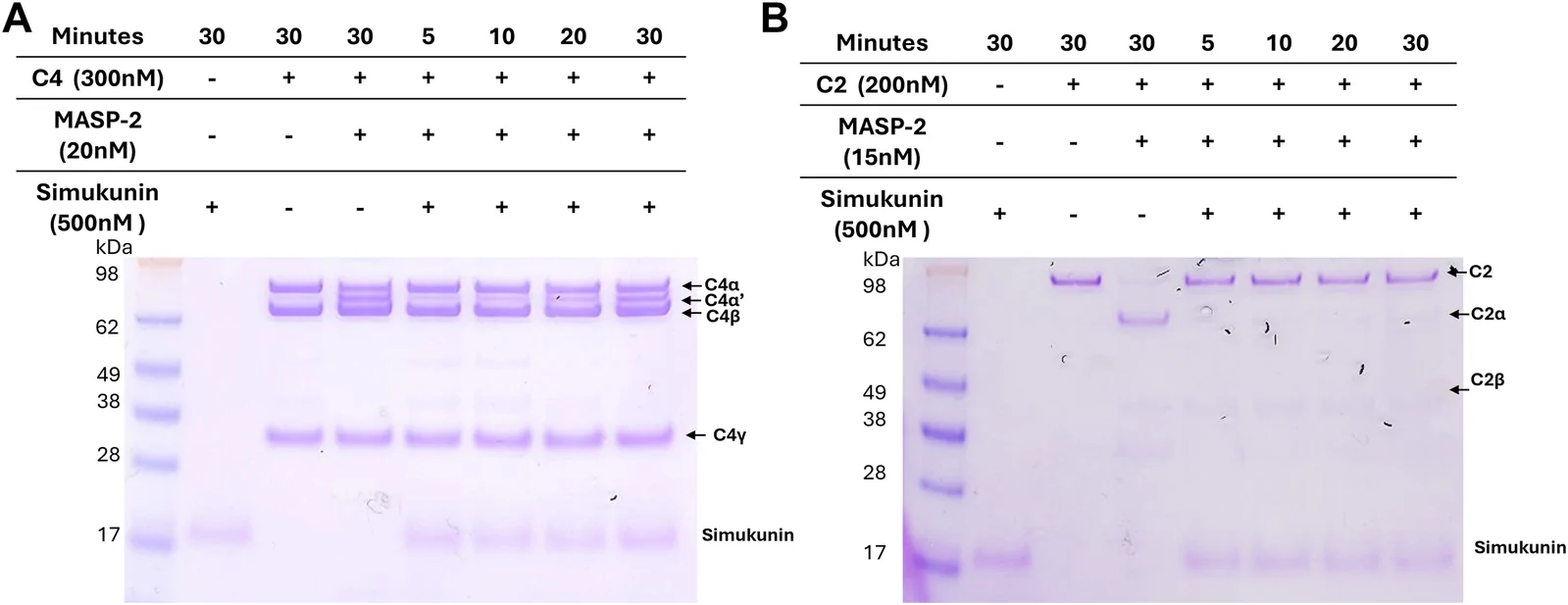
**Simukunin inhibits MASP-2–mediated cleavage of C4 and C2.***A*, C4 (300 nM) was incubated with MASP-2 (20 nM) and Simukunin (500 nM) to observe the presence of the indicated C4α′, C4β cleavage fragments. *B*, under similar conditions, C2 (200 nM) was incubated with MASP-2 (15 nM) in the presence or absence of Simukunin (500 nM) at 37 °C for 5, 10, 20, and 30 min. C2α and C2β cleavage fragments in the presence of Simukunin compared to MASP-2 alone are indicated. In all experiments, reaction products were resolved on 4 to 12% Bis-Tris gels under reducing conditions and visualized by Coomassie *Blue* staining.

### Simukunin does not inhibit cleavage of C1s or C1s proteolytic activity

When the CP is activated, C1r cleaves and activates C1s, driving the complement cascade amplification (18, 19). Because the SPR demonstrated residual binding to C1r, we tested if Simukunin could inhibit the cleavage of C1s proenzyme by binding to C1r. Moreover, because C1s and MASP-2 share molecular architectures and can cleave C2 and C4 (20, 21), we tested whether Simukunin could inhibit C1s activity. We incubated C1s proenzyme and C1r in the presence and absence of Simukunin. Then we incubated C2 or C4 with C1s and Simukunin. At 30 min, each reaction was stopped and loaded onto an SDS-PAGE gel followed by Coomassie blue staining. Simukunin did not inhibit the activity of C1r (Fig. 4*A*) nor C1s′ ability to cleave C2 and C4 (Fig. 4, *B* and *C*). The densitometric analysis for these experiments is shown in Figure S2. These results indicate that Simukunin specifically inhibits MASP-2.

**Figure 4:**
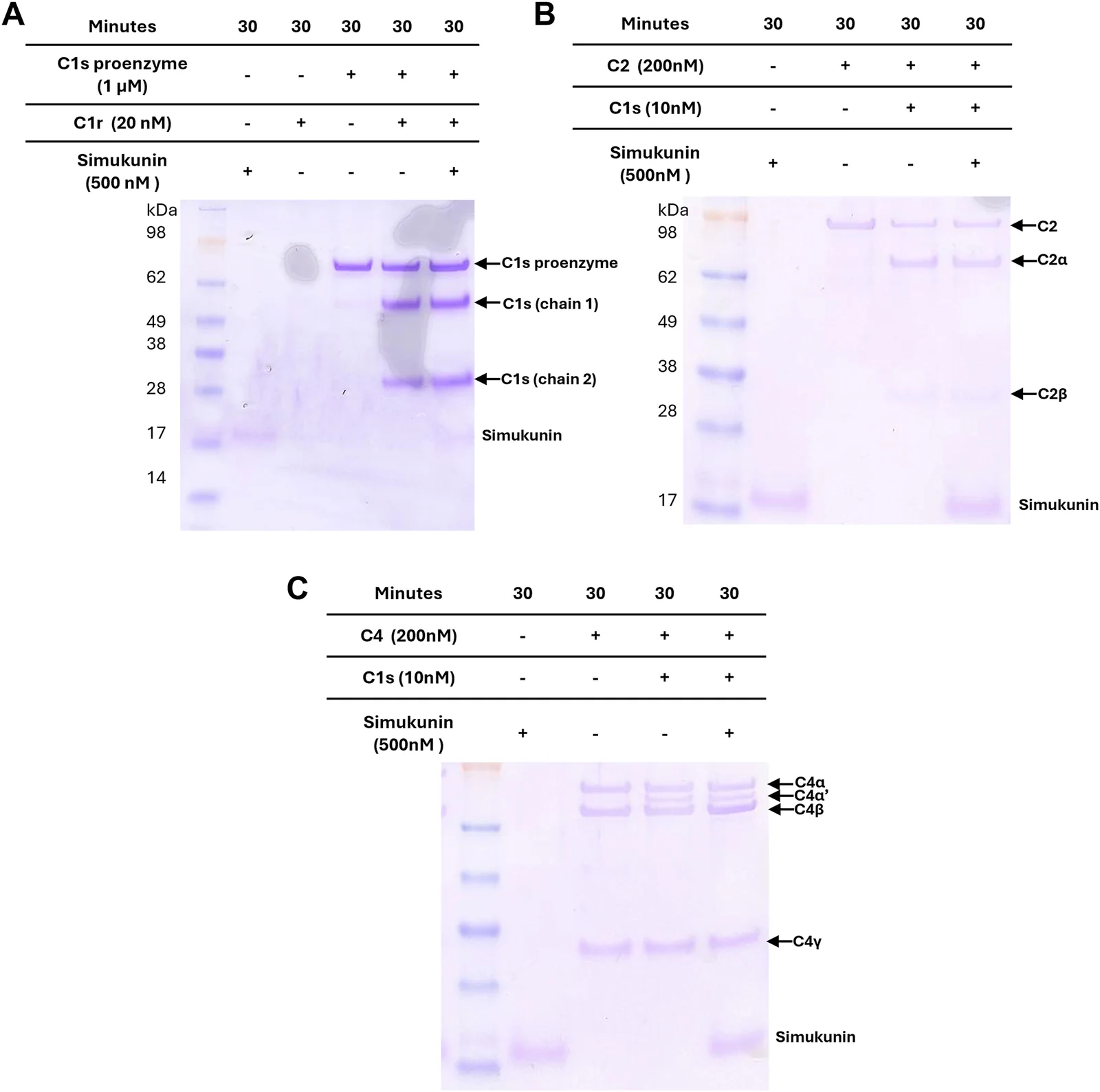
**Simukunin does not inhibit cleavage of C1s, C2, and C4.***A*, C1r (20 nM) was incubated with C1s proenzyme (1 μM) in the presence or absence of Simukunin (500 nM). Cleavage of C1s proenzyme by C1r was assessed after 30 min, and C1s cleavage products are indicated. *B*, C2 (200 nM) was incubated with C1s (10 nM) in the presence or absence of Simukunin (500 nM). C2α and C2β cleavage fragments are indicated. *C*, C4 (200 nM) was incubated with C1s (10 nM) in the presence or absence of Simukunin (500 nM). C4α′, C4β, and C4γ cleavage fragments are indicated. All experiments were incubated for 30 min at 37 °C and reaction products were resolved on 4 to 12% Bis-Tris gels under reducing conditions and visualized by Coomassie *Blue* staining.

### Simukunin inhibits thrombin formation by interacting with MASP-2

MASP-2 has been reported to cleave prothrombin to generate thrombin (22, 23). To further investigate the effects of Simukunin, we assessed thrombin formation mediated by MASP-2. Reactions were performed using MASP-2 and prothrombin in the presence or absence of Simukunin. Parallel assays with FXa were conducted as a control group. Thrombin formation was measured using chromogenic substrate S-2238. As expected, Simukunin reduced FXa-mediated cleavage of prothrombin (17). We also found that Simukunin significantly reduced generation of thrombin by MASP-2 (Fig. 5). These results confirm Simukunin can reduce generation of thrombin by inhibiting FXa and MASP-2.

**Figure 5:**
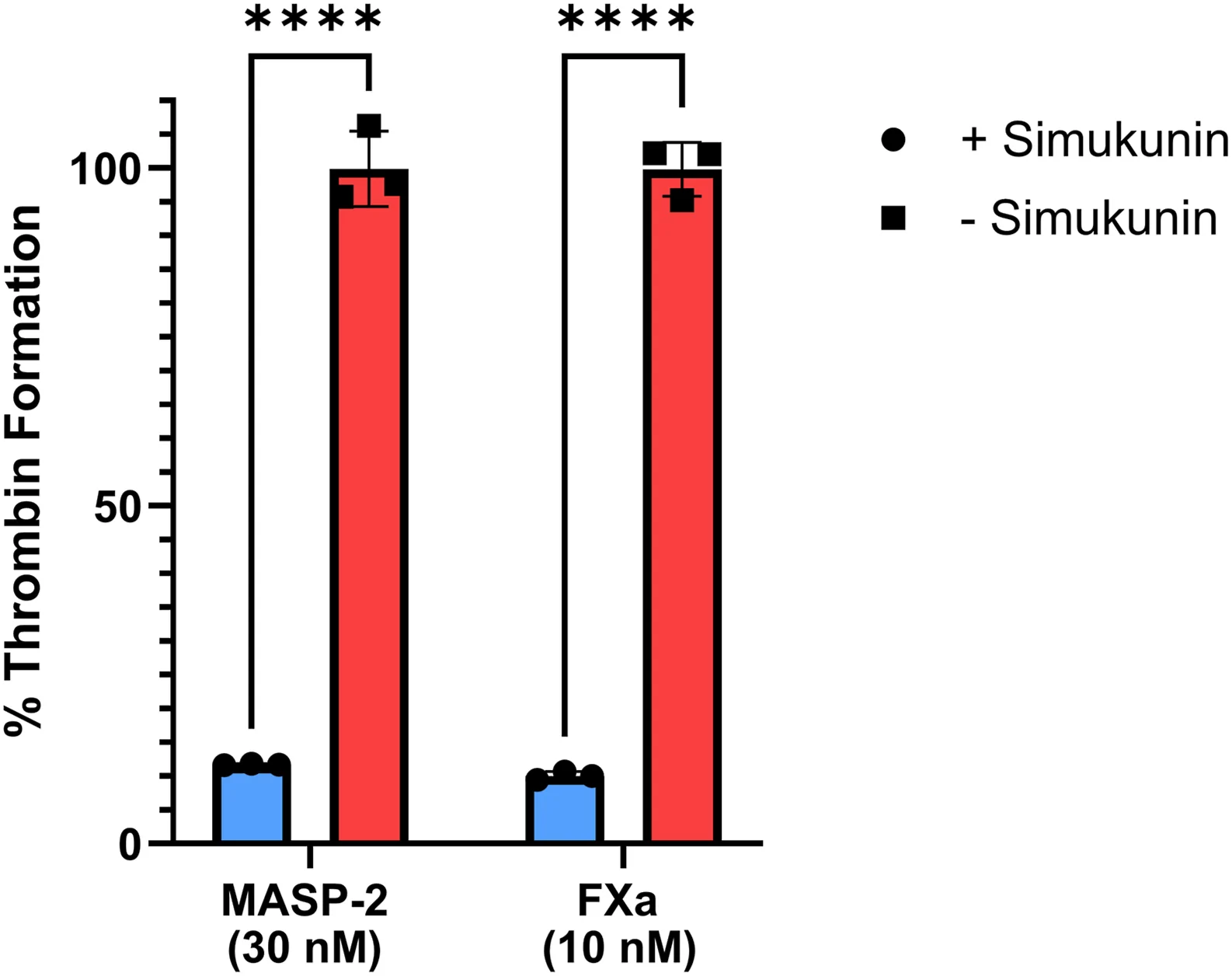
**Simukunin inhibits thrombin formation mediated by FXa and MASP-2.** Thrombin formation was assessed using the chromogenic substrate S-2238 at 5 mM with 10 nM prothrombin, 30 nM MASP-2 or 10 nM FXa, and 75 nM Simukunin. Absorbance was measured at 405 nm using a VersaMax microplate reader. Thrombin formation is expressed as a percentage of control (mean ± SD, n = 3 independent experiments). Statistical significance was determined using a two-tailed one-sample *t* test comparing SGE-treated samples with the normalized control value of 100%. ∗∗∗∗*p* < 0.0001 in GraphPad Prism 10.2.1.

### Simukunin improves survival in an LPS-induced sepsis in mice

To evaluate the immunomodulatory potential of Simukunin during acute systemic inflammation, we used an intraperitoneal LPS-induced sepsis model in BALB/c mice. Mice were treated with Simukunin 30 min before LPS injection, and survival was monitored for 48 h. Mice receiving LPS alone showed high mortality, with 85% succumbing in 48 h. In contrast, pre-treatment with Simukunin significantly improved survival compared with LPS group (Fig. 6). These results show that Simukunin has protective effects *in vivo* and has potential therapeutic applications.

**Figure 6:**
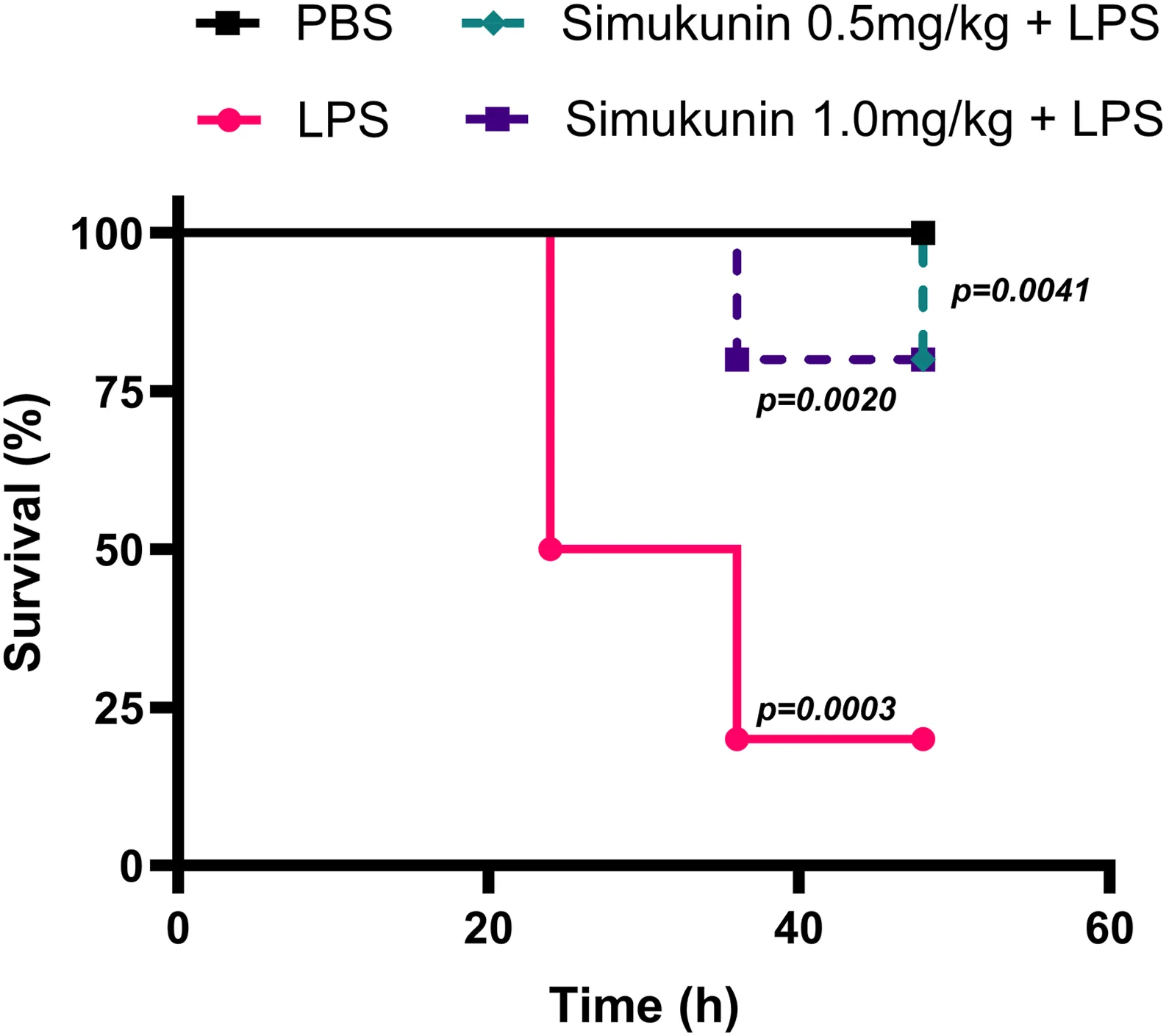
**Simukunin treatment improves survival in a murine model of LPS-induced sepsis.** Mice were inoculated with Simukunin (0.5 mg/kg or 1.0 mg/kg, i.p.) or PBS 30 min prior to LPS challenge (5 mg/kg, i.p.) and survival was monitored for 48 h (n = 10 per group). Statistical significance between survival curves was determined by the log-rank (Mantel-Cox) test; *p*-values are indicated in the figure.

### Immunolocalization of Simukinin in the salivary glands

Previous results with Simukunin demonstrate that this protein is constitutively expressed in the salivary glands of adult female *S. vittatum* and is not present in males (17). However, the immunolocalization of Simukunin was not evaluated. We assessed the immunolocalization of Simukunin in the salivary glands of female *S. vittatum* using polyclonal antibodies generated in mice against the recombinant protein. Our findings show that Simukunin is present in both the central lobe and the lateral lobes (Fig. 7).

**Figure 7:**
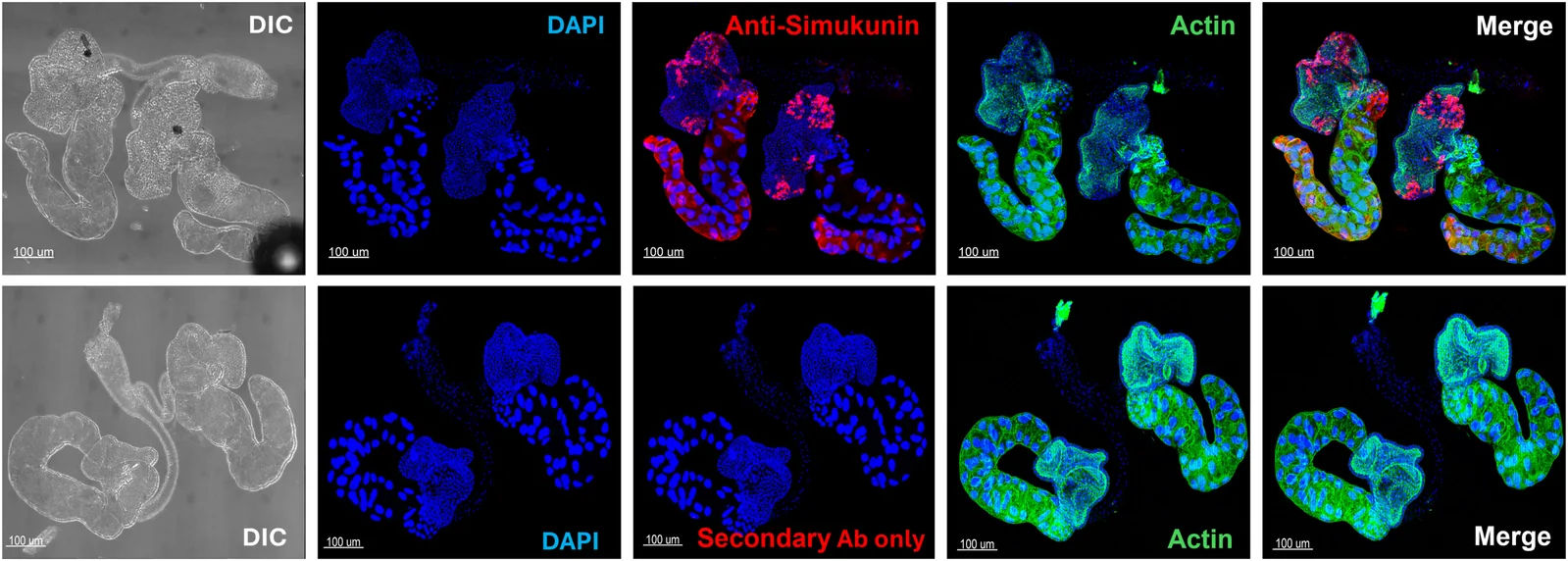
**Simukunin immunolocalization in female salivary glands.** Salivary glands dissected from adult *S. vittatum* female were fixed, permeabilized, and incubated with anti-Simukunin polyclonal primary antibodies, followed by a Texas Red-X-conjugated goat anti-mouse secondary antibody. The *upper panels* show representative salivary glands incubated with both the anti-Simukunin primary antibody and the fluorescently labeled secondary antibody. The *lowerpanels* show the negative control, in which the primary antibody was omitted, and glands were incubated with the secondary antibody (Ab) alone. Differential interference contrast (DIC) images are shown in *grayscale*. Nuclei were stained with DAPI (*Blue*), Simukunin (*Red*), and F-actin labeled with phalloidin Alexa Fluor 488 is shown in green. Merged images show the overlap of DAPI, Simukunin, and actin signals. Images were acquired using a Leica SP8 confocal microscope and processed using Imaris software.

## Discussion

Blood-feeding arthropods have evolved a range of anti-hemostatic molecules that enable successful feeding despite host defenses. In vertebrates, blood loss is controlled through coordinated responses including vasoconstriction, platelet aggregation, activation of the coagulation cascade, and complement signaling. To overcome these barriers, arthropods secrete saliva enriched with bioactive molecules that disrupt multiple points in these pathways. These molecules enable sustained blood flow at the bite site, facilitating efficient feeding and pathogen transmission (22, 23, 24, 25). The complement system comprises over 30 plasma proteins that circulate in the bloodstream in inactive forms. Upon activation, these proteins act in a coordinated cascade to form the MAC, which inserts pores into the membranes of invading pathogens, resulting in osmolysis (26, 27). By suppressing complement-mediated responses, salivary secretions of blood feeding arthropods maintain blood flow, minimize host detection, and may create a favorable environment for prolonged feeding. Anti-complement factors, such as Simukunin, may influence the fly survival and transmission of vector-borne pathogens, highlighting their importance in host–vector–pathogen interactions and their potential as targets for therapeutic or vector-control strategies.

In this study, we show that *S. vittatum* SGE strongly inhibits complement activation, with a pronounced effect on the LP, and identify Simukunin as the primary salivary factor responsible for this activity, with minimal impact on the CP and AP pathways. Our binding data provide mechanistic insight into this inhibition, demonstrating that Simukunin interacts strongly with MASP-2, while showing no detectable interaction with other complement components. Functionally, Simukunin preferentially inhibited MASP-2-mediated cleavage of C2 compared with C4, suggesting selective modulation of lectin pathway convertase formation. Although the structural basis underlying this substrate selectivity remains unclear, it may be influenced by differences in substrate recognition and affinity. One possible explanation is that stable recognition of C4 by MASP-2 makes C4 cleavage less susceptible to inhibition by Simukunin, whereas C2 processing may be more vulnerable to inhibition due to weaker enzyme–substrate interactions. Furthermore, while structural studies have elucidated the molecular basis of MASP-2 recognition and cleavage of C4, no structural information is currently available for the MASP-2–C2 complex, leaving the precise mode of C2 recognition unresolved (28, 29, 30, 31). Future structural and kinetic studies will be required to determine the molecular mechanism underlying Simukunin’s preferential inhibition of MASP-2-mediated C2 cleavage.

In contrast, Simukunin did not inhibit C1r or C1s activity, supporting its lack of effect on the CP. Extending these findings, Simukunin also suppressed MASP-2–mediated thrombin generation, mirroring its inhibitory effect on FXa and highlighting its impact on protease-driven inflammatory and coagulation pathways. Finally, in an LPS-induced sepsis model, Simukunin treatment modulated complement activation and improved survival, underscoring its functional relevance *in vivo.* LPS, a major component of Gram-negative bacterial cell walls, is a well-established activator of the lectin pathway, providing a mechanistic rationale for the protective effect of LP inhibition in this model. However, inflammatory biomarkers such as C3a, C5a, or cytokines were not measured in this study. The specific contribution of complement inhibition *versus* coagulation inhibition to the observed survival benefit remains to be determined in future studies.

Complement inhibitors have become increasingly relevant in managing inflammatory and thrombotic diseases because the complement system plays a central role in immune activation, endothelial injury, and coagulation (32, 33). Dysregulated complement activation contributes to a range of pathologies characterized by inflammation, microvascular injury, and thrombosis. In this context, complement inhibitors have emerged as an important therapeutic strategy, regulating inflammatory signaling and limiting immune-mediated tissue damage. For instance, Ravulizumab and Eculizumab are long-acting monoclonal antibodies that inhibit complement protein C5, preventing formation of the MAC and protecting the neuromuscular junction, which reduces muscle weakness in patients with Myasthenia Gravis (34, 35). Pegcetacoplan is a C3 inhibitor that blocks complement activation early, preventing downstream damage and protecting tissues in diseases such as paroxysmal nocturnal hemoglobinuria (36). More recently, inhibition of the lectin pathway at the level of MASP-2 has also shown therapeutic promise. In a mouse model of renal ischemia–reperfusion injury, the affinity-enhanced MASP-2 inhibitor EVO24L significantly reduced complement activation, inflammation, and tubular injury while preserving renal function, demonstrating that selective MASP-2 inhibition can effectively protect against complement-mediated tissue damage (37). These examples highlight the therapeutic potential of targeting the complement cascade at different points. However, currently available treatments are limited in number. As a result, there remains a significant unmet need for more effective and targeted anti-complement therapies.

To address this unmet need, we demonstrate that Simukunin can modulate the complement cascade, highlighting its potential as a candidate for targeted anti-complement therapy. Inhibition of the LP by Simukunin may enable *S. vittatum* to suppress local inflammation and facilitate blood feeding following skin penetration. Importantly, our results indicate that this effect is mediated through specific targeting of MASP-2, demonstrating a precise mechanism that may have potential applications in therapeutic intervention of complement activity. Taken together, these findings identify Simukunin as a promising therapeutic candidate for targeting complement-mediated immune activation and thrombosis.

## Experimental procedures

### Simukunin cloning, expression, and purification

The mature peptide of Simukunin was synthetized and subcloned by GenScript. The synthetic gene was codon-optimized for mammalian expression, engineered to contain 6x-His tag before the stop codon and subcloned into pHEK293 (Takara Bio). Human embryonic kidney Expi293 PRO (Thermo Fisher) cells were transfected with the respective plasmids at the Frederick National Laboratory for Cancer Research. Supernatants were collected 72 h post-transfection, centrifuged (100×*g*, 15 min), and frozen at −70 °C until purification. Simukunin purification was performed through affinity chromatography using HiTrap TALON crude columns (Cytiva). The protein was eluted with 20 mM Tris-HCl and 300 mM NaCl, pH 7.4. Chromatographic fractions were analyzed by SDS-PAGE, and the fractions containing the target protein were pooled and loaded on a Superdex 75 Increase 10/300 Gl (Cytiva).

### MASP-2 purification

The catalytic region of MASP-2 was expressed following the protocols described by Ambrus *et al.* (2003) and Dobó *et al.* (2008) (38, 39). Plasmids encoding the catalytic region of MASP-2 for bacterial expression (kindly provided by Peter Gal, Institute of Enzymology, Research Centre for Natural Sciences, Hungarian Academy of Sciences), were transformed into BL21 (DE3) pLysS competent cells. The transformed cells were then plated on Luria-Bertani (LB) medium containing 100 μg/ml ampicillin and 34 μg/ml chloramphenicol for selection. Protein expression was induced by adding 1 mM isopropyl-β-D-thiogalactopyranoside (IPTG) once the culture reached an optical density (OD600) of 0.6. After induction, cells were harvested and inclusion bodies were isolated by centrifugation. The inclusion bodies were then solubilized in a buffer containing 50 mM Tris-HCl, 6 mM reduced glutathione, 3 mM oxidized glutathione, 5 mM EDTA, and 0.75 M arginine at pH 9.0. The refolding process was carried out at 4 °C for 1 week. Following refolding, 1 mM benzamidine–HCl was added and maintained throughout all subsequent steps to prevent autoactivation of MASP-2. Refolding was completed by dialyzing the mixture against 25 mM MES and 0.5 mM EDTA, pH 9.0. MASP-2 was then purified by anion exchange chromatography using a HiPrep Q FF 16/10 column (GE Healthcare Life Sciences), with 1 mM benzamidine–HCl included during both dialysis and chromatography. The protein was eluted using a 0 to 300 mM NaCl gradient. Fractions were analyzed by SDS-PAGE, and those containing the target protein were pooled. The pooled protein was subsequently dialyzed against 25 mM Tris-HCl, 150 mM NaCl, pH 7.3 using Centricon filters (Thermo Scientific, MWCO 3 kDa).

### Salivary glands dissection

Blackflies were obtained through the BEI catalog number NR-53893, which is maintained and prepared for shipment by the University of Georgia. Salivary glands from adult female black flies were dissected in PBS (pH 7.4) using a stereomicroscope (Zeiss, Thornwood, NY, USA). Salivary gland homogenate (SGH) was prepared by sonicating dissected salivary glands (Branson Sonifier 450) in PBS pH 7.4. Disrupted tissues were then centrifuged at 12,000×*g* for 5 min, and supernatants were recovered. Protein concentration was measured by spectrophotometry at A_280_ nm (DS-11, DeNovix) and then stored at −80 °C until use.

### Production of polyclonal antibodies against Simukunin

Six-week-old female BALB/c mice (five per group) were inoculated with 50 μg of recombinant Simukunin in Magic Mouse adjuvant (Creative Diagnostics). Each animal received three immunizations at 21-day intervals. Four days after the last dose, terminal bleeds were performed. All injections and bleeds were carried out by NIAID/NIH Animal Facility staff. Serum from the five immunized animals per protein was pooled, stored at −30 °C, and analyzed by Western blot using the recombinant protein as a control.

### Western blot

Simukunin (2.5 μg/well) was separated by NuPAGE 4 to 12% Bis-Tris gel electrophoresis and transferred using the iBlotTM 2 PVDF Mini Stack System (iBlot, Invitrogen). The membrane was then blocked for 1 h at 37 °C with blocking buffer (5% (w/v) powdered nonfat milk in TBS). Polyclonal antibodies against Simukunin were diluted in blocking buffer (1:200) and incubated overnight. Goat anti-rabbit conjugated to alkaline phosphatase (Sigma cat. no.: A3687) was diluted in blocking buffer (1:5000) and used as the secondary antibody. Immunogenic bands were developed by adding the BCIP/NBT substrate (Promega). The reaction was stopped with distilled water.

### Immunofluorescence assays

Dissected salivary glands from *S. vittatum* were placed in shallow, concave wells of a 9-well plate (PYREX Spot Test Plates, 85 × 100 mm; well dimensions: 22 mm outer diameter × 7 mm deep; Thomas Scientific) and fixed with 4% paraformaldehyde (Sigma) for 30 min at room temperature. The tissues were then washed three times for 10 min each with PBS and blocked for 45 min using a solution containing 2% bovine serum albumin (BSA, Sigma), 0.5% Triton X-100 (Sigma), and PBS, pH 7.4. After washing three more times with PBS, the glands were incubated overnight at 4 °C with 200 μl of anti-Simukinin antibodies diluted 1:200 in 2% BSA, PBS. The primary antibodies were removed after three 10-min washes with 2% BSA, PBS, and the glands were then incubated with 2 μg/ml of Goat anti-Mouse IgG (H + L) Cross-Adsorbed Secondary Antibody, Texas Red-X (Thermo scientific) for 1 h in the dark at 4 °C. Excess secondary antibody conjugate was removed through three additional PBS washes. DNA was stained with 1 μg/ml DAPI (Sigma D9542), and actin was labeled with 0.04 μg/ml. Phalloidin Alexa 488 (Invitrogen) for 50 min. The glands were washed three times with PBS, placed on a glass slide, and mounted with a coverslip coated with 25 μl of Prolong Gold mounting medium (Invitrogen). Glands incubated with secondary-only controls were used to evaluate non-specific binding of the fluorescent secondary antibody. Slides were covered with aluminum foil, left to dry overnight at room temperature, and stored at 4 °C until imaging. Fluorescent images were taken using a Leica SP8 confocal microscope and processed with Imaris software version 9.2.

### The Wieslab assay for complement activation

Simukunin was evaluated using the commercial Wieslab Complement System Screen assay (COMPL300RUO) to assess the lectin, alternative, and classical pathways, following the manufacturer’s instructions. In these ELISA-based assays, normal human serum (NHS; Complement Technology) was diluted as specified in the kit’s protocol. Simukunin was then added to the diluted serum either at a fixed concentration of 1 μM or in serial dilutions, depending on the experimental design. The mixtures were incubated in microplate wells coated with MBL for the lectin pathway, human IgM for the classical pathway, or lipopolysaccharide (LPS) for the alternative pathway using Wieslab assay strips. Incubation was performed at 37 °C for 60 min. Complement activation was quantified by measuring optical density at 405 nm using a SpectraMax microplate reader (Molecular Devices), detecting the neoepitope of the terminal complement complex C5b-9. Deposition of C5b-9 reflects the level of complement pathway activity. Results were expressed as a percentage of activation relative to the positive control (100%), consisting of normal human serum alone, while the negative control (diluent only) was used to subtract background optical density values.

### Surface plasmon resonance (SPR) analysis

Surface plasmon resonance (SPR) experiments were performed on a T200 instrument (GE Healthcare Life Sciences) according to the manufacturer’s instructions. CM5 sensor chips, amine coupling reagents, and all buffers were obtained from GE Healthcare. HBS-P running buffer (10 mM HEPES, pH 7.4, 150 mM NaCl, and 0.005% (v/v) surfactant P20) was used for all measurements. Data were analyzed using Biacore Evaluation Software v2.0.3 (GE Healthcare). Briefly, Simukunin (15 μg/ml in 10 mM acetate buffer, pH 5.0) was immobilized on the CM5 sensor chip *via* standard amine coupling. Reference flow cells without immobilized protein were used for background subtraction. Binding assays were conducted at 25 °C with an association phase of 120 s at a flow rate of 30 μl/min, followed by a dissociation phase of 300 s. For kinetic analyses, the association time was extended to 180 s and the flow rate increased to 40 μl/min, with dissociation monitored for 600 s. The sensor surface was regenerated using a 45 s injection of Glycine, pH 2.0, at 30 μl/min between cycles. Kinetic parameters (*k*_on_, *k*_off_, and *K*_*D*_) were determined by global fitting of the sensograms using the 1:1 Langmuir binding model in the BIAcore Evaluation 3.1 software.

### Complement cleavage assays

MASP-2 (20 nM) was incubated with C4 (300 nM; Complement Technology), and in parallel, MASP-2 (15 nM) was incubated with C2 (200 nM; Complement Technology). All reactions were carried out in TBS-T buffer (20 mM Tris, 150 mM NaCl, 0.01% Triton X-100, pH 7.4) at 37 °C and monitored at 5, 10, 20, and 30 min in the presence or absence of Simukunin (500 nM). Where applicable, Simukunin was pre-incubated with MASP-2 for 10 min at 37 °C prior to substrate addition. Similarly, C1r (20 nM; Complement Technology) was incubated with C1s proenzyme (1 μM; Complement Technology) for 30 min at 37 °C under the same buffer conditions, either with or without Simukunin (500 nM). In inhibitor-containing reactions, C1r was pre-incubated with Simukunin for 10 min before addition of C1s proenzyme. In addition, C1s (10 nM; Complement Technology) was incubated with either C4 or C2 (200 nM; Complement Technology) in TBS-T buffer at 37 °C for 30 min, again in the presence or absence of Simukunin (500 nM), with a 10 min pre-incubation step where applicable. Following incubation, all reaction mixtures were resolved by SDS-PAGE using 4 to 12% NuPAGE gels under reducing and denaturing conditions and visualized by Coomassie staining. The gels were captured using the HP ScanJet 4580 scanner.

### Enzymatic assays

Experiments were performed as previously described by Szakacs *et al.* (2019) (40), with minor modifications. The IC_50_ value for the Simukunin–MASP-2 interaction was determined in a final assay volume of 100 μl in TBS-T using a 96-well microtiter plate and a VersaMax microplate reader. MASP-2 (20 nM) was pre-incubated with serial dilutions of Simukunin for 10 min at room temperature, followed by the addition of 250 μM Z-Lys-SBzl thioester substrate and 500 μM 5,5′-dithiobis (2-nitrobenzoic acid) as the co-substrate.

### Thrombin generation

Thrombin generation was assessed using the chromogenic substrate S-2238 (Diapharma). Reactions were carried out in a final volume of 100 μl in 25 mM HEPES, 150 mM NaCl, 5 mM CaCl_2_, and 0.05% BSA at pH 7.4. The assay mixture contained 5 mM substrate, 10 nM human prothrombin (Enzyme Research Laboratories), and 30 nM MASP-2, in the presence or absence of 75 nM Simukunin. For comparison, thrombin generation assays using human FXa (Enzyme Research Laboratories), were performed under identical buffer conditions, with 10 nM FXa and 75 nM Simukunin. Absorbance at 405 nm was monitored using a VersaMax microplate reader.

### LPS-induced sepsis in mice

Female BALB/c mice (strain code 028) were purchased from Charles River Laboratories. All animal procedures were conducted in accordance with the Public Health Service Animal Welfare Assurance (#A4149-01) and approved by the NIH Office of Animal Care and Use Committee (OACUC), under protocol ID ASP-LMVR3. Mice were divided into four groups (1): PBS control (2), LPS only (5 mg/kg) (3), LPS + Simukunin (0.5 mg/kg), and (4) LPS + Simukunin (1 mg/kg). PBS and Simukunin were administered intraperitoneally at a final volume of 100 μl. After 30 min, the mice were intraperitoneally challenged with LPS (Sigma) at the concentrations described above. Survival was monitored for 48 h.

## Data availability

Data are available upon request.

## Supporting information

This article contains supporting information.

## Conflict of interest

The authors declare that they have no conflicts of interest with the contents of this article.
