# Supplementary material for: The black fly salivary protein Simukunin inhibits complement activation through the lectin pathway protease MASP-2
Source: J Biol Chem. 2026 Sep 4;302(10):113529. doi: 10.1016/j.jbc.2026.113529 (PMC13656542; doi:10.1016/j.jbc.2026.113529)
Supplement: Supplementary file 1 [file mmc1.docx]

**Figure S1: Purification of Simukunin.** (A) Simukunin was expressed and purified as described in the Materials and Methods section. Size-exclusion chromatography (SEC) on a Superdex 75 Increase 10/300 GL column served as the final purification step. (B) Coomassie-stained 4-12% Bis-Tris gel electrophoresis of the collected SEC fraction. (C) Simukunin (25μg/well) was tested by Western blot using anti-Simukunin at a 1:200 dilution, and Goat anti-rabbit conjugated to alkaline phosphatase (1:5,000) was used as the secondary antibody. Immunogenic bands were developed by adding the BCIP/

NBT substrate (Promega), as shown.

**Figure S2:** Densitometric analysis of complement protein cleavage assays. Cleavage products from the experiments shown in Figures 3 and 4 were quantified by densitometry. For Figure 3, the indicated C4α′ (A) and C2α (B) cleavage products generated by MASP-2 were quantified in the presence or absence of Simukunin at different time points. For Figure 4, the indicated C1s chain 1 generated by C1r-mediated activation of C1s (C), and the C2α (D) and C4α′ (E) cleavage products generated by C1s were quantified in the presence or absence of Simukunin after 30 min. Band intensities were determined from Coomassie Blue-stained gels using ImageJ 1.52n. For all gels, band intensities in the presence of Simukunin were expressed as a percentage relative to the corresponding condition without Simukunin, which was set to 100%. Data are presented as mean ± SD from three independent experiments. Statistical significance was determined using two-way ANOVA followed by Dunnett’s multiple comparisons test for panels A and B, and an unpaired *t*-test for panels C–E.*P < 0.05; **P < 0.01; ***P < 0.001; ****P < 0.0001.
